# Identification of Optimal Reference Genes for Normalization of qPCR Analysis during Pepper Fruit Development

**DOI:** 10.3389/fpls.2017.01128

**Published:** 2017-06-29

**Authors:** Yuan Cheng, Xin Pang, Hongjian Wan, Golam J. Ahammed, Jiahong Yu, Zhuping Yao, Meiying Ruan, Qingjing Ye, Zhimiao Li, Rongqing Wang, Yuejian Yang, Guozhi Zhou

**Affiliations:** ^1^State Key Laboratory Breeding Base for Zhejiang Sustainable Pest and Disease Control, Institute of Vegetables, Zhejiang Academy of Agricultural SciencesHangzhou, China; ^2^Suzhou Polytechnic Institute of AgricultureSuzhou, China; ^3^Department of Horticulture, Zhejiang UniversityHangzhou, China

**Keywords:** qPCR, reference gene (RG), normalization, pepper, fruit development

## Abstract

Due to its high sensitivity and reproducibility, quantitative real-time PCR (qPCR) is practiced as a useful research tool for targeted gene expression analysis. For qPCR operations, the normalization with suitable reference genes (RGs) is a crucial step that eventually determines the reliability of the obtained results. Although pepper is considered an ideal model plant for the study of non-climacteric fruit development, at present no specific RG have been developed or validated for the qPCR analyses of pepper fruit. Therefore, this study aimed to identify stably expressed genes for their potential use as RGs in pepper fruit studies. Initially, a total of 35 putative RGs were selected by mining the pepper transcriptome data sets derived from the PGP (Pepper Genome Platform) and PGD (Pepper Genome Database). Their expression stabilities were further measured in a set of pepper (*Capsicum annuum* L. var. 007e) fruit samples, which represented four different fruit developmental stages (IM: Immature; MG: Mature green; B: Break; MR: Mature red) using the qPCR analysis. Then, based on the qPCR results, three different statistical algorithms, namely geNorm, Normfinder, and boxplot, were chosen to evaluate the expression stabilities of these putative RGs. It should be noted that nine genes were proven to be qualified as RGs during pepper fruit development, namely *CaREV05* (CA00g79660); *CaREV08* (CA06g02180); *CaREV09* (CA06g05650); *CaREV16* (Capana12g002666); *CaREV21* (Capana10g001439); *CaREV23* (Capana05g000680); *CaREV26* (Capana01g002973); *CaREV27* (Capana11g000123); *CaREV31* (Capana04g002411); and *CaREV33* (Capana08g001826). Further analysis based on geNorm suggested that the application of the two most stably expressed genes (*CaREV05* and *CaREV08*) would provide optimal transcript normalization in the qPCR experiments. Therefore, a new and comprehensive strategy for the identification of optimal RGs was developed. This strategy allowed for the effective normalization of the qPCR analysis of the pepper fruit development at the whole pepper genome level. This study not only explored the optimal RGs specific for studying pepper fruit development, but also introduced a referable strategy of RG mining which could potentially be implicated in other plant species.

## Introduction

Over the last several decades, pepper (*Capsicum annuum* L.) has become an economically important vegetable all over the world. Unlike tomato plants, which are a typical respiratory climacteric fruit (Colombie et al., 2016), pepper fruit development has drawn the attention of researchers, due to its representative non-respiratory climacteric properties (Lee et al., 2014). During the course of pepper fruit development, a number of complex developmental processes and regulatory pathways have been determined to contribute to the overall changes in fruit size, texture, and other substance compositions (Klee and Giovannoni, 2011; Gómez-García and Neftalí Ochoa-Alejo, 2013; Ruiz-May and Rose, 2013).

The recent technical progress which has been made regarding physiological and molecular tools has enabled the effective elucidation of the complicated biological processes which occur during fruit development. For instance, quantitative real-time PCR (qPCR) is a frequently used biological means for elucidating the molecular mechanisms of the genes of interest which are involved in various biological processes, such as the carotenoid and capsaicin metabolisms in pepper fruit (Curry et al., 1999; Ginzinger, 2002; Sung et al., 2005). In qPCR experiments, the gene expressions can be quantified by normalization with one or more of the stably expressed internal reference genes (RGs) (Pfaffl et al., 2004; Huggett et al., 2005). This process can remove the non-biological variations caused by such factors as the different amounts and quality of the starting material, variable enzymatic efficiency of the reverse transcription, or sample differences in the overall transcriptional activity (Suzuki et al., 2000; Bustin et al., 2005; Exposito-Rodriguez et al., 2008). Accordingly, it is known that the stabilities of the internal RGs are critical for reliable and accurate qPCR results (Huggett et al., 2005; Gutierrez et al., 2008; Guénin et al., 2009).

Generally speaking, an optimal RG should be defined as a gene which is stably expressed among various tissues, and under different experimental treatments (Czechowski et al., 2005; Huggett et al., 2005; Exposito-Rodriguez et al., 2008). However, as demonstrated in the results of some recent studies, several well-known and frequently used RGs have been proven to be inappropriate for normalization due to their expression variabilities under certain conditions (Czechowski et al., 2005; Jain et al., 2006; Exposito-Rodriguez et al., 2008; Gutierrez et al., 2008; Jian et al., 2008; Remans et al., 2008; Jarosova and Kundu, 2010; Mascia et al., 2010; Wang et al., 2012). In practical terms, the expression levels of most RGs have been determined to be dependent on specific conditions, including tissue types, developmental stages, and experimental set-ups. Therefore, no single RG has been found to be widely applicable under various experimental conditions. Moreover, it has been well recognized that at certain times a single RG may not be adequate for accurate normalization in gene expression analyses (Yoo et al., 2009; Wang et al., 2012). Therefore, a systematic valuation of RGs should be conducted prior to their use in specific qPCR analysis in order to achieve more reliable results (Bustin et al., 2009; Guénin et al., 2009).

To date, several stable RGs have been specifically identified for the qPCR analyses of fruit development in different plant species, such as watermelon (Li et al., 2012; Kong et al., 2015), papaya (Zhu et al., 2012), and blueberry (Die and Rowland, 2013). However, even though considerable attention has been given to the molecular mechanism of pepper fruit development (Jang et al., 2015; Liu et al., 2017), the RGs in pepper fruit have not yet been characterized. Only a small number of the previous related studies focused on the identification of the optimal RGs for qPCR analysis under various stress conditions, or in different pepper tissues, and these studies were mainly based on the evaluation or validation of some previously reported candidate RGs (for example, house-keeping genes) under various conditions (Wan et al., 2011; Wang et al., 2012). Currently, due to the completion of the genome sequencing of pepper (*C. annuum* L.; Kim et al., 2014; Qin et al., 2014), there is now an opportunity to re-identify the best RGs for normalization under different conditions within the entire genome level. Therefore, considering the high complexities of fruit developmental processes, this study's aim was to identify some novel optimal RGs for future qPCR analyses of pepper fruit development, based on the availability of the pepper genome database (Kim et al., 2014; Qin et al., 2014).

In this study, the expression stabilities of all of the pepper (*C. annuum* L.) genes during the various fruit developmental stages were initially evaluated, based on the published RNA-seq data (Kim et al., 2014; Qin et al., 2014). A total of novel stably expressed genes were identified as putative RGs, and were further validated through a qPCR technique. Then, using three different statistical algorithms (geNorm, Normfinder, and Boxplot) which had been designed to evaluate the expression stabilities of the genes (Vandesompele et al., 2002; Andersen et al., 2004; Pfaffl et al., 2004), 10 optimal genes were identified as qualified RGs for normalization during different stages of the pepper fruit development (IM, MG, B, and MR). Moreover, in accordance with the geNorm algorithm, the combined use of the two top-ranked RGs (*CaREV05* and *CaREV08*) could potentially improve the reliability of the qPCR results. When taken together, and based on the availability of the entire pepper genome, the optimal RGs for pepper fruit developmental study were comprehensively identified and evaluated through large-scale biological information mining, and qPCR techniques. The results not only provided useful and referable RG resources for the accurate studies of gene expressions in the fruit development of pepper and other non-climacteric plants, but also shed light on the effective identification system for the best RGs under various plant conditions.

## Materials and methods

### Collection and evaluation of the previously reported RGs

**Figure 1:**
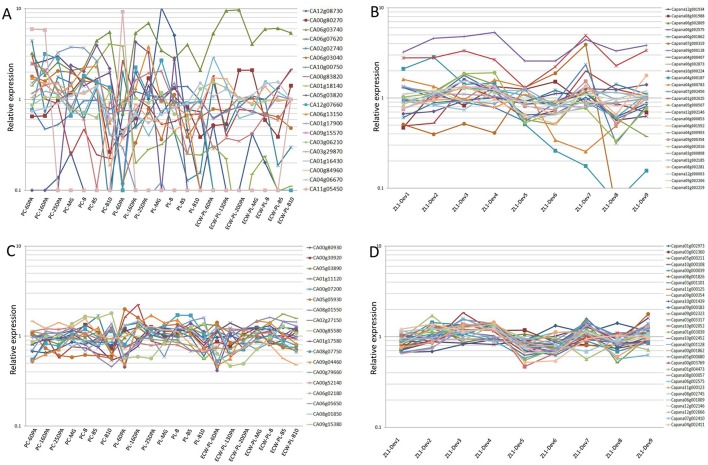
Relative expression of the previously reported RGs **(A,B)**, and novel RGs **(C,D)** over the different pepper fruit developmental stages, based on the RNA-seq data. The relative expression levels per gene were derived from the RNA seq data, and calculated by dividing the expression value (RPKM value) by the average expression level calculated across all of the samples of the various pepper fruit developmental stages.

In this study, the pepper RGs reported by the previous research studies (Wan et al., 2011; Wang et al., 2012) were selected in order to evaluate the expression stabilities during the stages of pepper (*C. annuum* L.) fruit development based on the RNA-seq data (http://peppergenome.snu.ac.kr/; http://peppersequence.genomics.cn/). Furthermore, the orthologous genes of 5 and 12 RGs which had been identified during the fruit development stages in tomato and watermelon (Coker and Davies, 2003; Kong et al., 2015), respectively, were also used to evaluate the stability of the gene expression in pepper. The details, including the accession number and gene description, are listed in Table 1. The corresponding gene sequences of these candidate RGs were then collected from the NCBI (National Center for Biotechnology Information: https://www.ncbi.nlm.nih.gov/); TFGD (Tomato Functional Genomics Database: http://ted.bti.cornell.edu/); and CuGenDB (Cucurbit Genomics Database: http://www.icugi.org/), respectively. Then, using a Blastn search, the orthologous genes (*E*-value set at 1e^−5^) were collected in two pepper genome databases: the Pepper Genome Platform, PGP-http://peppergenome.snu.ac.kr/; and the Pepper Genome Database, PGD-http://peppersequence.genomics.cn/, as shown in Table 1. In this study, based on the RNA-seq data which had been previously reported (Kim et al., 2014; Qin et al., 2014), the Reads Per Kilobase Million (RPKM) value of each of the orthologous genes was collected. Also, the average expression in the different fruit developmental stages were calculated (Supplemental Table 1). The relative expression levels per gene were obtained by dividing the expression values of all the development stages of the fruit by the calculated average expression (Figures 1A,B).

**Table 1:** Homologous genes of the previously identified RGs derived from the PGP (Pepper Genome Platform) and PGD (Pepper Genome Database).

**Gene bank**	**Gene locus (PGP)**	**Gene locus (PGD)**	**Gene description**	**Identities (PGP) (%)**	**Identities (PGD) (%)**
GQ339766	CA12g08730	**Capana12g001934**	Actin gene	100	100
AY572427	CA00g80270	Capana08g001988	Actin mRNA	99	100
GQ365708	CA06g03740	Capana06g002809	PPR1 protein	100	99
AF242732/ AY496125	CA06g07620	Capana06g002575	Translation elongating factor 1a/Elongation factor 1-alpha	97	97
EU401723	CA08g18890	Capana00g001862	Cyclophilin	100	100
AJ246009	CA02g02740	Capana07g000319	Glyceraldehyde-3-phosphate dehydrogenase	99	99
AY484392	CA09g18320	Capana09g000128	Mitochondrial substrate carrier family protein	99	99
DQ924970	CA04g20140	Capana04g000407	Ubiquitin-conjugating enzyme family protein	99	100
AY486137	CA06g03040	Capana06g002873	Ubiquitin-conjugating protein	99	100
AJ246013.1	CA10g00750	Capana10g000224	GAPCP-2, NAD bingding, Glyceraldehyde-3-phosphate dehydrogenase	100	100
EF495259.1	**CA04g21850**	**Capana04g000187**	β-tublin	99	99
EF495257.1	CA00g83820	Capana04g000783	TUA6, structural constituent of cytoskeleton, protein bingding	99	99
TC123837	CA07g21150	Capana07g002456	Phosphoglycerate kinase	93	93
TC115713	CA01g18140	Capana01g002025	Chaperonin-60 beta chain prec	94	94
TC123959	CA05g03820	Capana05g000507	UBI-3-like protein	91	91
TC124053	CA12g07660	Capana12g002146	UBI-3-like protein	94	94
TC123964	CA06g20620	Capana06g000853	AT5g	91	91
Cla016178	CA06g13150	Capana06g001552	Clathrin adaptor complex subunit	99	100
Cla021905	CA01g17900	Capana04g000903	Protein phosphatase 2A regulatory subunit A	83	83
Cla012277	CA09g15570	Capana09g000354	A member of RANGTPase gene family	83	83
Cla021565	CA03g06210	Capana06g002016	Cytosolic ribosomal protein S15	83	84
Cla001870	CA03g29870	Capana03g000808	SAND family protein	83	83
Cla011119	CA01g16430	Capana01g002185	TATA binding protein 2	82	82
Cla016074	CA08g17190	Capana08g002281	TIP41-like family protein	87	87
Cla003129	CA12g22930	Capana12g000003	Alpha tubulin 5	82	82
Cla022418	**CA04g21850**	**Capana04g000187**	β-tublin	84	84
Cla017746	CA00g84960	Capana09g002306	Ubiquitin-protein ligase 7	80	80
Cla007792	CA04g06670	**Capana12g001934**	β-actin	86	86
Cla010159	CA11g05450	Capana01g002219	18SrRNA	81	83

*The common genes derived from different homologous are labeled in bold (for example: **CA04g21850**, **Capana12g001934**)*.

### Selection and validation of the stably expressed genes in the RNA-seq data sets

In this research study, by utilizing the entire sets of RNA-seq data files (Kim et al., 2014; Qin et al., 2014), the genes with RPKM values between 200 and 2,000 (medium expressions) at all of the development stages of the fruit were selected (Supplemental Table 2). The CV (co-efficient variation) value of each gene was calculated (Supplemental Table 2), and the genes with CV ≤ 0.35 were chosen as the putative RGs in the following qPCR analysis (Table 2).

**Table 2:** Primer sequences and PCR amplification characteristics of the 35 selected RGs.

**Gene name**	**Gene ID**	**Gene description**	**Primer sequence (5′−3′)**	**Product size (bp)**	**E**	***R*^2^**
*CaREV01*	CA00g52140	Polyadenylate-binding protein 8-like	F: GCAAGGTCAACGTCCAGGTG	132	0.91	0.992
			R: AGGCCACGTCCTGGAGGATA			
*CaREV02*	CA08g01850	Calcyclin-binding protein-like	F: GTGTGCTTCAGTGCCATCTT	68		
			R: GGTTACGGATCACCTTTGCT			
*CaREV03*	CA09g15380	Transcription factor LUX-like	F: AGTCCACAAGCAACAACAGC	148	0.91	0.996
			R: ATGGTGCATGGCTAGTTGAA			
*CaREV04*	CA02g27150	Protein COBRA-like	F: GCTGGACGTGGGCAAAGAAG	140		
			R: TCCAGGCATCATGTCAACGACT			
*CaREV05*	CA00g79660	Polyubiquitin-like	F: GGACCAGCAAAGGTTGATTT	94	1.05	0.997
			R: CAGATGGAGGGTTGATTCCT			
*CaREV06*	CA08g07750	Proline iminopeptidase	F: GGTACTCCATTCCCGACCT	144	0.89	1
			R: AATGAAGAACAACGGGAACC			
*CaREV07*	CA05g05930	unknown	F: TGATTGAGGAATGCGGGTCACT	96	0.84	0.923
			R: TAGCAAGAGCATCCGCCACT			
*CaREV08*	CA06g02180	Putative late blight resistance protein	F:CCTCGGGAATCTAGAAATCTTGCATGT	56		
			R: AGCACGAGTTGCTCTAATGCTCT			
*CaREV09*	CA06g05650	Uncharacterized protein	F: TTGTGAGGCAAACAAGAGGA	95	0.96	0.999
			R: TGAATGAACCAAACCCTCA A			
*CaREV10*	CA09g04460	Uncharacterized protein	F: GGGATGGCCGCATTAGCATC	118	1.7	0.994
			R: GGTGCATGAATGGGCATGGA			
*CaREV11*	Capana08g002745	SKP1-like protein 1B	F: GCTGTTGCTTTGGAATCTCA	100	1.06	0.978
			R: TGGACAGGATCTTGCTGGTA			
*CaREV12*	Capana09g002323	60S ribosomal protein L24	F: TGGGTGCAACCTTGGAGGTA	150	0.94	0.982
			R: TGGCCATCACTTCTGCCTTCT			
*CaREV13*	Capana10g002052	Ubiquitin-conjugating enzyme E2	F: GACTCTCGGTTCAGGAGGAG	127	0.84	0.999
			R: ATCTCCATCATCCATCCCAT			
*CaREV14*	Capana05g000211	Uncharacterized	F: CGATCATGAAATCTCAGCGT	133	0.92	0.997
			R: TGCTGCTTCAATTTCTCCAC			
*CaREV15*	Capana07g002410	Heat shock cognate protein 80	F: CAACCAGAGCTCTTCATCCA	109	0.99	0.995
			R: TACCCAGGTTGTTCACCAGA			
*CaREV16*	Capana12g002666	Histon H3.3	F: GACTGATCTGCGTTTCCAGA	139	1.03	0.989
			R: TTGAATGTCCTTGGGCATAA			
*CaREV17*	Capana10g002452	Uncharacterized	F:ACATGCAACAGTTTGAGTTTCCACA	188	0.9	0.941
			R: TGGGACGTCCGATAAACGCA			
*CaREV18*	Capana00g001862	Peptidyl-prolyl cis-trans isomerase	F: GTCGTGATGGAGCTGTTCGC	90	0.71	0.994
			R: CATCCTTCCGACGCCCTTCT			
*CaREV19*	Capana01g003039	CBS domain-containing protein	F: CGTCACACCTGAAACCAAAG	150	0.98	0.997
			R: TCTTCCCTGTGCTCACTCAC			
*CaREV20*	Capana03g003317	Probable histon H2A	F: GCAGGAAAGACAACAGCAGCAG	101	0.95	0.998
			R: GGAAACTGGAGACCTGCACGA			
*CaREV21*	Capana10g001439	Ras-related protein RABI1a-like	F: ACATCAGGAATTGGATCCGT	112	0.97	0.996
			R: GATGTAGGAACAGCCCGTTT			
*CaREV22*	Capana03g002360	Memberane steroid-binding protein 2	F: TGGTGAAGCTAAGCCAACAG	110	0.97	0.996
			R: CGTCACCATCAGACTTGTCC			
*CaREV23*	Capana05g000680	Uncharacterized	F: TTAAACCACTCCGGTTCTCC	144	0.99	0.999
			R: TCTTGTCGTCGGAGAGATTG			
*CaREV24*	Capana00g000039	Peptidyl-prolyl cis-trans isomerase	F: GAGCCATCTACTGTGGCTCA	110	0.94	0.998
			R: ACAGCTTTGGGTCTGGAAAC			
*CaREV25*	Capana12g002146	CBS domain-containing protein CBSX3	F: TTGCCCAAGCTTATGATGTC	145	1.06	0.992
			R: CCACCACCAAAGAATGATTG			
*CaREV26*	Capana01g002973	Probable histon H2A	F: ATCTGCAGAAGCACCAGTTG	68	0.98	0.991
			R: CACGCAGCGTTAATTCAAGT			
*CaREV27*	Capana11g000123	Ras-related protein RABE1a-like	F: CTGTTGGTCGTGTTGAAACC	143	0.99	0.997
			R: GAAACCGACATTGTCACCAG			
*CaREV28*	Capana10g000108	Membrane steroid-binding protein 2	F: GGGAGCCGGTGGTAATTCGT	135	0.86	1
			R: CAGCCCTGTTTGAACCAGCA			
*CaREV29*	Capana08g000057	Coiled-coil domain-containing protein 9	F: GGGATGCTGTGCTGCTTGTT	133	0.86	1
			R: GTTGCACAAGTGCTCTGGATGT			
*CaREV30*	Capana00g003769	Nascent polypeptide-associated complex subunit alpha-like protein 1	F: ACAACCGCAACCACAACCAC	114	0.87	0.998
			R: CCGTAACATTGGTCGCAGCA			
*CaREV31*	Capana04g002411	UBI-3 protein homolog	F: GTGCTGCTCAGACCAAGAAG	148	0.73	0.989
			R: CCAACAGCAGCAACAGATTT			
*CaREV32*	Capana06g001009	Iron-sulfur cluster assembly protein 1	F: GTGGTGCACCTCAAGACAAC	137	0.93	0.999
			R: CACTGTTGGTGGCTTATTGG			
*CaREV33*	Capana08g001826	Elongationfactor 1-alpha-like	F: AGAGGCATGCGAAGCTGTCA	119	0.79	0.999
			R: GATGCTGAGCCCAGACCGT			
*CaREV34*	Capana01g004473	Uncharacterized	F: ACGCCTAGCGTTCATTCGGT	124	0.83	0.999
			R: GACGCCGTGATTCTGCCTTC			
*CaREV35*	Capana11g000125	ABC transporter F family member 1	F: AAACCAGGTGGCCCATGAGA	112	0.8	0.995
			R: TTCACCCAATCCGGCCCTTT			
No CDS	CA00g85580	Unknwon				
No CDS	CA01g17580	Unknwon				
No CDS	CA00g30920	Unknwon				
No primer	CA05g03890	Unknwon				
No primer	CA01g11120	Protein cornichon homolog 4-like				
No primer	CA00g07200	Ubiquitin-conjugating enzyme E2				
No primer	CA00g80930	GTP-binding protein				
No primer	CA08g01550	Putative F-box protein				
No primer	Capana03g001101	Ubiquitin-conjugating enzyme E2-like				
No primer	Capana09g000354	GTP-binding nuclear protein Ran-3				
No primer	Capana09g000183	Nucleosome assembly protein 1				
No primer	Capana03g001128	Iron-sulfur cluster assembly protein 1				
No primer	Capana06g002575	Elongation factor 1-alpha				

### Plant materials used in this study

In this study's experiments, the pepper (*C. annuum* L.) inbred line “007e” was used. Its fruit characteristically become fully mature (red flesh) at approximately 60 days after pollination. The 4-week-old seedlings used in this study were transplanted to a greenhouse at the Zhejiang Academy of Agricultural Sciences, Hangzhou, China (east longitude 120°2′, north latitude 30°27′) for the experimental process. Also, this study's field management was implemented following the standard commercial practices. The pepper fruit were harvested at the four developmental stages as follows: Immature (IM); Mature Green (MG); Breaker (B); and Mature Red (MR). Three fruit were randomly collected at each sampling point, and each of these represented a biological replication. Then, all of the samples were flash-frozen in liquid nitrogen, and stored at −80°C until used in the subsequent experiments.

### Total RNA isolation and cDNA synthesis

The total RNA from all of the samples was isolated using the TRIZOL reagent from the frozen samples, in accordance with the manufacturer's protocol (Tiangen, Beijing, China). The concentration and purity of the extracted RNA were measured using a BioDrop ULite spectrophotometer (Biochrom, England). The RNA samples with A260/A280 > 1.8 and A260/A230 > 2.0 (which indicated good RNA quality) were used in this experiment. All of the RNA samples were adjusted to the same concentration in order to ensure that the RNA input was homogenized for subsequent reverse transcription reactions. Then, in accordance with the manufacturer's instructions (TIANGEN, Beijing, China), the genomic DNAs (gDNA) were eliminated from the RNA samples, and the single-stranded cDNAs were synthesized.

### Primer design and qPCR analysis

In this study, gene-specific primers were designed using a Real-time PCR (TaqMan) primer design (http://www.genescript.com; the primer details are listed in Table 2). A qPCR analysis was performed in a 96-well plate using an SYBR Green-based PCR assay. A 20 μL reaction mixture, which contained 6 μL of diluted cDNA (10 ng); 10 μL of SYBR Green PCR Master Mix (Invitrogen, USA); 250 nM of each primer; and 0.1 μL of ROX, was prepared. The mixture was subjected to the following program: 10 min at 94°C; 30 cycles of 45 s at 94°C; 45 s at 55°C; and 1 min at 72°C, following a 7-min extension at 72°C (ABI real-time PCR system, USA). There were three technical duplications performed for all of the reference genes. At this point, melting curves were created and exhibited for all the investigated qPCR products (Supplemental Figure 1). The amplified products were resolved on 1.5% agarose gel (Supplemental Figure 2). The amplification efficiency (E) and correlation coefficient (*R*^2^) for each of the genes were calculated using a standard curve method (Table 2).

### Evaluation of the expression stability

The expression levels of the tested genes were obtained through the qPCR experiments, and the results were depicted as Ct values (Supplemental Table 4). A Boxplot was drawn using the stock chart in Excel 2007 in order to show the expression variations of each gene. The difference between whisker-up limit and floor limit (Whisker *D*-value) in the Boxplot was then calculated, as shown in **Figure 3**. The amplification efficiency (E) was calculated with the following formula: E = (10^−1/slope^−1), and the slope was generated by amplifying the 10-fold serial dilution of the cDNA samples. As described previously, geNorm and NormFinder software packages were used in this study to evaluate the gene expression stability (Vandesompele et al., 2002; Andersen et al., 2004). The geNorm applet provided a measure of the gene expression stability (M), and created a stability ranking via the stepwise exclusion of the least stable genes. In other words, the genes with the lowest M values were the genes with the highest expression stability. In addition, the geNorm also provided the pairwise variation values (V) for the determination of the least number of RGs required for reliable normalization. No additional genes were required for normalization when the pairwise variation (Vn/n + 1) was below 0.15 (Vandesompele et al., 2002). The NormFinder approach has been developed to measure the variations across groups, and to determine the expression stabilities of the tested genes (Andersen et al., 2004). The stability values acquired from the Boxplot, geNorm and NormFinder are listed in Supplemental Table 5.

### Determination of the *CaPAL* expression and capsaicin content levels during the development of pepper fruit

This study's samples were collected from pollinated “007e” pepper fruit at 30 dpp (days past pollination), 40, 50, and 60 dpp, respectively. The expression pattern of the *CaPAL* gene (Capana04g000187) is known to be associated with the accumulation of capsaicin in pepper fruit (Curry et al., 1999; Perucka and Materska, 2001). The primer sequences for the *CaPAL* gene are F: 5′-GGTCCCAATGGTGAGAAACTTAATGC-3′ and 5′-AACAGGACCATCGACGCCAT-3′. These sequences were designed according to the previously mentioned methods. The two top-ranked RGs identified in this study (*CaREV05* and *CaREV08*), as well as *CaUBI-3* (Capana06g002873, a previously identified RG in pepper) (Wan et al., 2011), were used for the normalization. The relative expression levels of the *CaPAL* gene were calculated based on a 2^−ΔΔCt^ algorithm. The normalization factor of two RG combinations was calculated using the geometric mean. Then, fresh pepper fruit at four different developmental stages (IM, MG, B, and MR) were collected for the RNA extraction, and the subsequent gene expression analysis. The extraction and measurement of the capsaicin content levels were conducted according to the previously described methods (Sung et al., 2005). Three biological replicates were adopted in the qPCR analysis and capsaicin content level measurements.

## Results

### Evaluation of the previously identified RGs during the development of the pepper fruit

In this study, two pepper (*C. annuum* L.) genome databases which had been obtained by different research groups (PGP: Pepper Genome Platform, http://peppergenome.snu.ac.kr/; and PGD: Pepper Genome Database, http://peppersequence.genomics.cn/), (Kim et al., 2014; Qin et al., 2014) were used for the analysis. Then, based on the previously reported RGs for pepper (Wan et al., 2011; Wang et al., 2012), 12 paralogous genes (identities > 97%) from the PGP and PGD, respectively, were identified (Table 1). Moreover, this study collected five previously reported candidate RGs for tomato fruit (Coker and Davies, 2003), and 12 candidate RGs for watermelon fruit (Kong et al., 2015), for a total collection of 17 orthologous genes in each of the databases (PGP and PGD) (Table 1). Among the identified homologs of the reported RGs, three genes (CA04g21850 in the PGP; and Capana12g001934, Capana04g000187 in the PGD) were found to be redundant. Therefore, a total of 45 pepper homologous genes of RGs (28 from the PGP; 27 from the PGD) were collected in the present study. The detailed information regarding these RGs was acquired from NCBI, TFGD, and CuGenDB (see details in the materials and methods section), and is listed in Table 1.

In the RNA-seq data derived from the PGP, the RPKM values of these 28 RGs, in seven different developmental stages of the fruit (6DPA, 16DPA, 25DPA, MG, B, B5, and B10), from the pericarp and placenta of two accessions (*C. annuum* L. Var. CM334 and *C. annuum* L. Var. ECW), were used to evaluate their expression stabilities (Supplemental Table 1). Among these, eight genes (CA08g18890, CA09g18320, CA04g20140, CA04g21850, CA07g21150, CA06g20620, CA08g17190, and CA12g22930) were not available for further evaluation, due to the incomplete RNA-seq data obtained from the PGP (Supplemental Table 3). Similarly, for the RNA-seq data of the PGD, the expression levels of the 27 genes in nine fruit developmental stages of the *C. annuum* L. Var. Zunla-1 were demonstrated (Supplemental Table 1). Then, based on the RPKM values of these 47 reported RGs (20 from the PGP, 27 from the PGD), this study analyzed the relative expression of all of the genes among the different developmental stages of the pepper fruit (Supplemental Table 2). In regard to the 20 reported RGs collected from the PGP, most of these (17/20) demonstrated high expression variabilities (CV > 0.35) during pepper fruit development (Figure 1A; Supplemental Table 3). Some of the CV values were even higher than 1.5 (CA12g08730 [2.41] and CA05g03820 [1.53]), which indicated that these genes displayed poor expression stabilities during the pepper fruit development. In regard to the 27 reported RGs collected from the PGD, it was determined that 10 of these showed unqualified variations (CV > 0.35) as RGs for the fruit developmental analysis (Figure 1B). Further analysis demonstrated that, among the remaining 17 stably expressed reported RGs, most of these (13/17) had average expression levels (RPKM values) of less than 200 (Supplemental Table 1), which indicated that they were not qualified for normalization due to their low expression levels. It was determined that only the PPKM values of four RGs, i.e., Capana06g002575 (668.6), Capana00g001862 (802.4), Capana12g002146 (1298.6), and Capana09g000354 (404.9), were high enough (>200) to be considered as candidate RGs (Supplemental Table 1). Therefore, this study concluded that the majority of the previously reported RGs were not well qualified for normalization in the different developmental stages of the pepper fruit.

### Identification of the putative RGs based on RNA-seq data analysis

In the present study, a strategy was developed to identify the corresponding candidate RGs, and to evaluate their expression stabilities during the different stages of pepper fruit development. A total of 74 genes with RPKM values ranging from 200 to 2,000 during the various stages of the fruit development were identified from the PGP and PGD, respectively, by searching all the sets of RNA-seq derived data (Supplemental Table 2). The CV values of these 74 genes were then calculated. It was found that more than half (48/74) of the CV values were lower than 0.35 (Supplemental Table 3). These 48 genes, including 18 genes from the PGP, and 30 genes from the PGD, are detailed in Table 2. Further analysis revealed that the genes identified from the entire genome level were more stable than those previously reported candidate RGs during the different developmental stages of the pepper fruit (Figure 1).

### qPCR analysis of the putative RGs identified during the pepper fruit development

The intention of this study was to validate the expression stability of the 48 genes as RGs for pepper fruit developmental study using a qRCR analysis. The results showed that the gene sequences of three candidate RGs (CA00g85580, CA01g17580, and CA00g30920) were missing due to the incomplete genome database information. Also, the proper primers of 10 candidate RGs (for example, CA05g03890 and Capana03g001101) for the qPCR analyses could not be designed due to their short cDNA sequences, or their high homologies with other genes in the pepper plants. Therefore, a total of 35 candidate RGs, which were designated as *CaREV01* to *CaREV35*, were eventually chosen for the further expression validation in the qPCR analysis (Table 2).

As shown in Table 2, the amplicon lengths of the 35 candidate RGs ranged from 68 bp (*CaREV02*) to 198 bp (*CaREV08*). A qPCR amplification was carried out with specific primers based on the mRNA sequences of the 35 candidate RGs, as listed in Table 2. The melting curve analysis showed a single product peak (Supplemental Figure 1), which meant that no non-specific amplicons were observed in the absence of the template amplification. Furthermore, the PCR-amplification specificities of the 35 primer pairs were verified by agarose gel electrophoresis using cDNA templates (Supplemental Figure 2). The amplification efficiencies (E) of these candidate RGs were found to vary from 0.71 (*CaREV18*) to 1.7 (*CaREV10*), and more than half of the primer pairs (18/35) ranged from 0.9 to 1.1, which indicated optimal primer pairs (Table 2). However, the amplification efficiencies (E) of three of the genes (*CaREV02, CaREV04*) could not be calculated, due to their low transcript level in the pepper fruit (Table 2; Supplemental Table 4). The correlation coefficients (R^2^) of the 35 candidate RGs ranged from 0.941 (*CaREV17*) to 1 (*CaREV06, CaREV28*, and *CaREV29*), as shown in Table 2.

**Figure 2:**
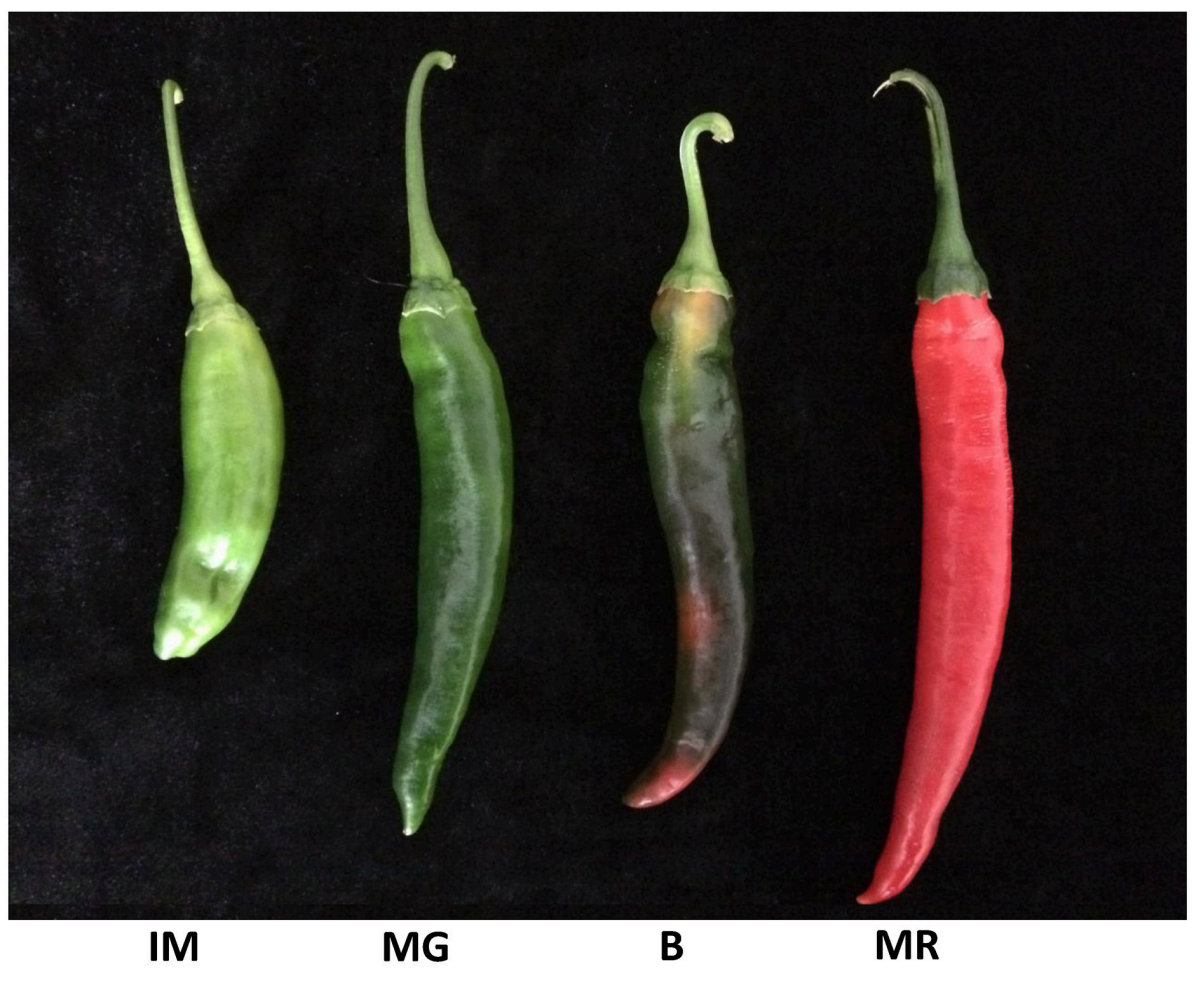
Pepper fruit samples from four representative developmental stages: IM, Immature, 30 days after fertilization; MG, Mature green, 40 days after fertilization; B, Breaker, 50 days after fertilization; MR, Mature red, 60 days after fertilization.

The Ct values of each of the putative RG derived from four pepper fruit developmental stages (IM, MG, B, and MR) were used in this study to evaluate the expression levels (Supplemental Table 4; Figure 2). The average Ct values of the majority of the candidate RGs (32/35) in the various developmental stages of the fruit ranged between 20 and 30 (From 21.71 [*CaREV18*] to 29.08 [*CaREV11*]). The remaining three candidate RGs were *CaREV02* (32.27), *CaREV04* (32.45), and *CaREV10* (31.36) (Supplemental Table 4).

### Validation of the selected RGs based on the boxplot, geNorm and normfinder results

For the boxplot analysis, 12 of the 35 candidate RGs were identified for low whisker difference values (Whisker *D*-value). These candidates were: *CaREV16, CaREV21, CaREV27, CaREV24, CaREV09, CaREV28, CaREV33, CaREV31, CaREV05, CaREV08, CaREV23*, and *CaREV32* (Table 3; Figure 2). Therefore, based on the boxplot standard, these 12 candidate RGs were defined as the most stably expressed. The geNorm analysis determined that the 12 most stable candidate RGs with average expression stabilities (M) less than 0.92 were: *CaREV05, CaREV08, CaREV31, CaREV33, CaREV09, CaREV27, CaREV21, CaREV16, CaREV28, CaREV23, CaREV26*, and *CaREV32* (Table 3). This study also used Normfinder to evaluate the stability of these 35 putative RGs. 12 of the RGs were identified as being stably expressed (stability value less than 0.7), according to the statistical algorithm of Normfinder, i.e., *CaREV16, CaREV21, CaREV27, CaREV26, CaREV14, CaREV23, CaREV19, CaREV28, CaREV09, CaREV20, CaREV25*, and *CaREV32*.

**Table 3:** Most stable RGs evaluated according to the Boxplot, geNorm, and NormFinder, respectively.

**Boxplot**	**Whisker *D*-value**	**geNorm**	***M*-value**	**NormFinder**	***M*-value**	**Stability rank**
CaREV16	0.951	CaREV05	0	CaREV16	0.192	1
CaREV21	0.990	CaREV08	0	CaREV21	0.357	2
CaREV27	1.107	CaREV31	0.097	CaREV27	0.446	3
CaREV24	1.443	CaREV33	0.230	CaREV26	0.482	4
CaREV09	1.592	CaREV09	0.323	CaREV14	0.497	5
CaREV28	2.023	CaREV27	0.402	CaREV23	0.580	6
CaREV33	2.371	CaREV21	0.449	CaREV19	0.582	7
CaREV31	2.426	CaREV16	0.553	CaREV28	0.583	8
CaREV05	2.552	CaREV28	0.640	CaREV09	0.634	9
CaREV08	2.552	CaREV23	0.776	CaREV20	0.678	10
CaREV23	2.572	CaREV26	0.859	CaREV25	0.697	11
CaREV32	2.586	CaREV32	0.910	CaREV32	0.699	12

*The stability values were evaluated with three different statistical algorithms. The expression stability of each RG decreases from top to bottom*.

Although the three different assessing systems (Boxplot, geNorm, and Normfinder) provided different results, 12 of the putative RGs (*CaREV05, CaREV08, CaREV31, CaREV33, CaREV09, CaREV27, CaREV21, CaREV16, CaREV28, CaREV23, CaREV26*, and *CaREV32*) were found to occur in at least two different statistical algorithms (Table 3). Generally speaking, the Ct value of a suitable RG should be between 15 and 30. Also, the optimal amplification efficiency (E) of primer pairs should be between 0.9 and 1.1 (Tiangen, China). Among these 12 genes, it was found that the average Ct value of *CaREV23* was closer to the upper limit (28.4) (Supplemental Table 4), and the amplification efficiency (E) of the *CaREV28* primers was less than 0.9 (0.86) (Table 2). Therefore, the authors of this study do not recommend these two genes as RGs. The remaining 10 putative *CaREV*s, including *CaREV05, CaREV08, CaREV31, CaREV33, CaREV09, CaREV27, CaREV21, CaREV16, CaREV26*, and *CaREV32*, were identified as being qualified RGs for normalization in pepper fruit development.

Recently, some research studies have reported that the application of more than one RG in normalization can assist in obtaining more reliable qPCR results (Reid et al., 2006; Exposito-Rodriguez et al., 2008; Gutierrez et al., 2008). Therefore, the present study utilized geNorm software to calculate the pairwise variation values (V) between two sequential normalization factors which contained an increasing number of genes (see the materials and methods section). The pair-wise variation revealed that the V2/3 value was 0.05 (significantly <1.5), which suggested that the combined use of the two top-ranked RGs, i.e., *CaREV05* and *CaREV08*, would suffice for an improved normalization in pepper fruit developmental studies (**Figure 5**).

### Practical validation of the top-ranking RGs: *CaREV05* and *CaREV08*

In this study, in order to practically validate the reliability of the two top-ranked RGs (*CaREV05* and *CaREV08*), the expression patterns of the *CaPAL* and capcaisin contents were analyzed in the fruit of the “007e” pepper line at four developmental stages (IM, MG, B, and MR). *CaREV05* and *CaREV08*, as well as their combination (*CaREV05*/*CaREV08*), were used to normalize the expressions of the *CaPAL*. Meanwhile, *CaUBI-3*, which was the previously identified RG in the pepper (Wan et al., 2011), was used as the control. As presented in Supplemental Figure 3, the transcript abundance of *CaPAL* at the IM stage of the pepper fruit development was set as the control. Then, by using the *CaREV05, CaREV08*, or *CaREV05*/*CaREV08* combination for the normalization, the similar *CaPAL* expression trends were observed. The following were the observational results: First, a sharp increase occurred after the IM stage; a peaking occurred at the MG stage; and then a decreasing trend was observed. Therefore, the expression levels at the MG stage were at least 25 times higher than those at the IM stage (Supplemental Figure 3). Meanwhile, the highest expression level was observed using the single RG-*CaREV08* (34.07 times), and the lowest expression level was observed using the single *CaREV05* (25.59 times). Also, moderate expression levels were found using the paired *CaREV05*/*CaREV08* (29.83 times). However, when the CaUBI-3 was used for the normalization, a distinct *CaPAL* expression pattern was observed, in which no sharp changes in the expression were evident among all the developmental stages of the pepper fruit (Supplemental Figure 3). The capsaicin accumulation was also measured in the pepper fruit during the four developmental stages (IM, MG, B, and MR). The results showed that the capsaicin content gradually increased with the ripening of the fruit, and reached the highest level (11.84 mg.g^−1^ dry weight) at the B stage, which was followed by a gradual decrease (Supplemental Figure 3). It is known that the capsaicin biosynthesis is closely correlated with the expression of the *CaPAL* gene (Sung et al., 2005). In this study, this correlation was noticeable when the *CaREV05, CaREV08*, or *CaREV05*/*CaREV08* was used as the RGs for the normalization. However, when normalized with the *CaUBI-3*, no such close correlations were detected.

## Discussion

The advent of qPCR technology has revolutionized the gene expression analysis in recent years. However, accurate qRCR results are mainly dependent on the use of stable RGs for normalization, which have the ability to minimize the non-biological variations of different samples. Therefore, the identification of proper RGs is essential for obtaining reliable data in qPCR analyses (Udvardi et al., 2008; Bustin et al., 2009; Guénin et al., 2009). Currently, some previously identified RGs, such as “House Keeping Genes” (for example, Actin, Ubiquitin, and 18s rRNA), are frequently used across a broad range of tissue samples, and under various experimental conditions (Bustin, 2002; Kong et al., 2014). However, an increasing amount of evidence has shown occurrences of high variations in these widely-used RGs under various experimental conditions, or in different assayed organs (Guénin et al., 2009; Warzybok and Migocka, 2013). Therefore, the selection of suitable RGs for specific conditions is critical, in order to avoid unnecessary errors in the qRCR experimental results.

Pepper fruit is viewed as a typical non-respiratory climacteric fruit. Therefore, studying its development has important reference value for other plants of the same type (Nielsen et al., 1991; Aza-González et al., 2012; Martínez-López et al., 2014; Cheng et al., 2016), including blackberry, cherry, and cucumber. Furthermore, the capsaicin biosynthesis that occurs only in the fruit of capsicum plants has always been a hot research topic (Curry et al., 1999). To date, mainly two research groups have been conducting research regarding the optimal RG identification and evaluation in pepper, and some of the applicable RGs were selected in the current study (Wan et al., 2011; Wang et al., 2012). Furthermore, previous studies have shown that beta tubulin (β-TUB), ubiquitin-conjugating protein (UBI-3), and the elongation factor 1-alpha (EF1α), are optimal RGs under abiotic stresses (osmotic stress, cold, and heat), as well as hormonal treatments (salicylic, abscisic, and gibberellic acids). Moreover, it was reported that ‘UBC10 (UBI-3), glyceraldehydes-3-phosphate dehydrogenase (GAPDH), and Actin gene (Actin1) were the most stably expressed among the different tissues (leaves, stems, roots, flowers, fruit, and seeds) (Wan et al., 2011; Wang et al., 2012). However, despite all of the applicable RGs which had been validated in pepper, none of these RGs had yet been validated during the pepper fruit development. In the present study, the relative expressions of these previously identified RGs were analyzed, and the results indicated their instabilities during different stages of the pepper fruit development (Figures 1A,B). This indicated the necessity to identify some novel RGs for the normalization of the qPCR analyses in the pepper fruit studies.

To date, many studies have been conducted to validate appropriate RGs through evaluations of the expression stabilities of traditional RGs under specific conditions (Czechowski et al., 2005; Lovdal and Lillo, 2009; Schmidt and Delaney, 2010; Dekkers et al., 2012). In regard to the pepper fruit development, this study collected nearly 60 homologous genes of the candidate RGs which had already been reported in previous publications (Coker and Davies, 2003; Wan et al., 2011; Wang et al., 2012; Kong et al., 2015). Then, their corresponding expression stabilities were validated during the various stages of fruit development according to the RPKM values derived from the RNA-seq data sets. Surprisingly, the majority of these candidate RGs were not well qualified for normalization as internal control genes, due to either their low transcript levels, or unstable expressions (Table 1; Figure 1). Therefore, it was necessary for the research team to develop a new strategy to identify the optimal RGs for pepper fruit developmental studies.

**Figure 3:**
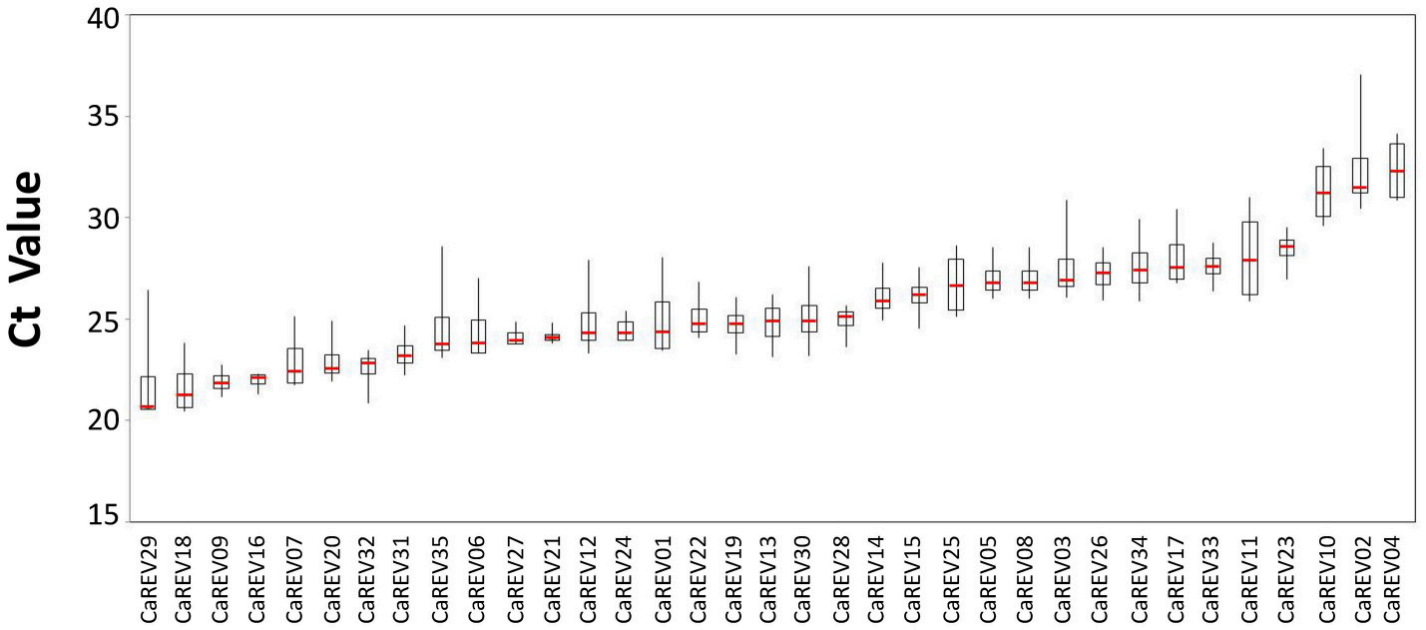
Boxplot analysis of the expression stability of the tested RGs during four pepper fruit developmental stages. The Boxplot figure was drawn using the stock chart in Excel 2007, and the difference between the whisker up-limit and floor-limit (Whisker *D*-value) in the Boxplot was calculated based on the stock chart algorithm. The line shown in the box is the median value. The whisker caps represent the minimum and maximum values.

**Figure 4:**
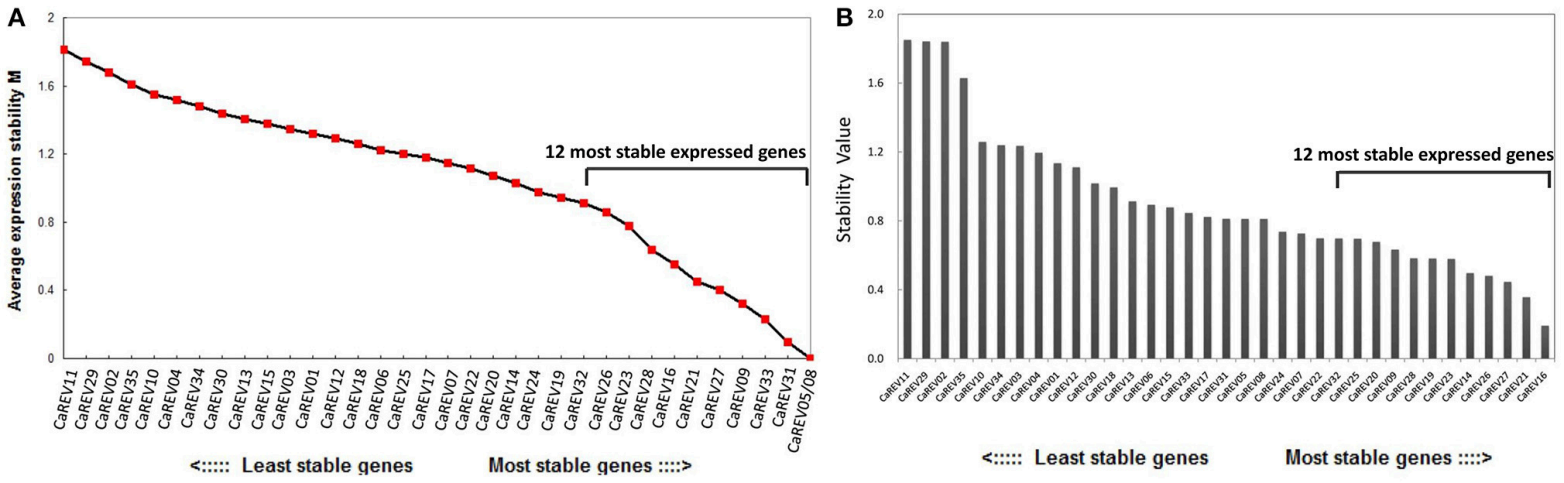
Expression stability of the newly identified candidate RGs analyzed by the geNorm **(A)** and NormFinder **(B)**. **(A)** The ranking is based on the principle that the gene expression ratio between the two ideal internal control genes is identical in the various samples. The expression stability values (M) of the 35 candidate RGs are shown, and the 13 most stable expressed genes are labeled; **(B)** The Norm Finder is a model-based approach which evaluates the expression variations, and then ranks the RGs with minimal estimated intra-group variations. The 13 most stably expressed genes are labeled in the figure.

The availability of the pepper (*C. annuum* L.) genome databases (Kim et al., 2014; Qin et al., 2014) allowed us to search for genes which were stably expressed on the entire genome level. Therefore, an entire genome level screening was conducted based on the RNA-seq data sets. Initially, 35 different pepper genes were collected as putative RGs for fruit developmental studies (Table 2). Then, further validations were conducted by qPCR analyses in different pepper fruit samples, including IM (Immature), MG (Mature Green), B (Break), and MR (Mature Red) (Figure 2). To date, many algorithms have been designed to assist in the selection of optimal RGs. Among these, geNorm and NormFinder are two well-known statistical algorithms for RG validation (Zhu et al., 2012). In this study, the geNorm determined that 12 genes were the best RGs (Figure 4A; Table 3). Also, according to the Normfinder, 12 genes, including *CaREV16, CaREV21*, and *CaREV27*, were validated as being relatively better RGs (Figure 4B). Furthermore, this study found that the whisker difference values in the Boxplot were referable for the evaluation of the expression variations. For example, *CaREV16, CaREV21*, and *CaREV27* were the top three ranked RGs according to the whisker *D*-value in the Boxplot, which was exactly the same as demonstrated in the Normfinder (Figure 3; Table 3). It should be noted that variations in the validation results were expected due to the different algorithms which were adopted in the three methods (Vandesompele et al., 2002; Andersen et al., 2004). Therefore, by considering the above-mentioned evaluation results of the three algorithms, along with the other referable factors (gene transcript level, primer amplification efficiency), 10 genes (*CaREV05, CaREV08, CaREV31, CaREV33, CaREV09, CaREV27, CaREV21, CaREV16, CaREV23*, and *CaREV26*) were identified as suitable candidate RGs for fruit development studies.

**Figure 5:**
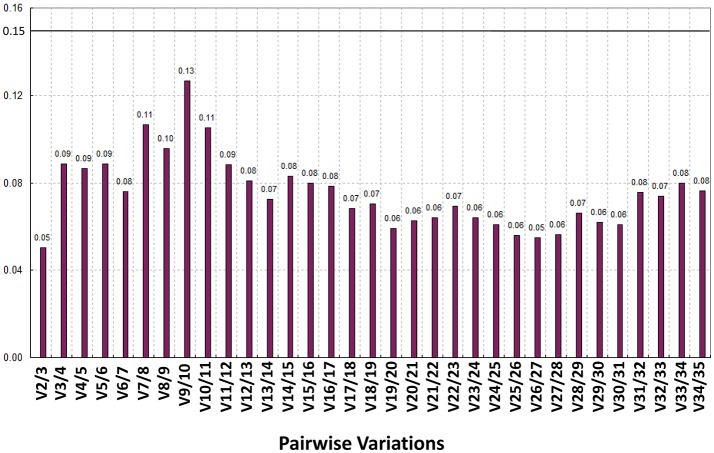
Analysis of the best RG association using the geNorm algorithm. The pairwise variations (*V*-values) were calculated in the geNorm. When Vn/n+1 was less than 0.15, the n gene was qualified as an RG association; V2/V2 was 0.05 (<0.15), which meant *CaREV05* and *CaREV08* qualified as an RG combination.

In general, it is known that the application of more than one RG in the normalization can efficiently improve the accuracy of the qPCR results (Reid et al., 2006; Exposito-Rodriguez et al., 2008; Gutierrez et al., 2008). Therefore, in order to explore the appropriate number of RGs in the pepper fruit development, the V values based on the geNorm were calculated in the present study (Figure 5). According to the geNorm evaluation, a combination of two RGs (*CaREV05* and *CaREV08*) would be a better choice for normalization when more reliable qPCR results were required.

Notably, capsaicin is a symbolic substance in pepper fruit. Fortunately, the regulatory mechanism of capsaicin biosynthesis has been clearly elucidated (Curry et al., 1999; Perucka and Materska, 2001; Narasimha Prasad et al., 2006). Moreover, *CaPAL* is one of the key genes which regulates capsaicin accumulation in pepper fruit (Curry et al., 1999; Perucka and Materska, 2001). Therefore, the correlation between the *CaPAL* expression pattern and capsaicin biosynthesis during the development of pepper fruit has been effectively used for reliable evaluations of the top-ranked RGs. According to the results of Sung et al. (2005), a high expression level of *CaPAL* can lead to the initial stage of capsaicin accumulation. Therefore, good correlations were found between the capsaicin accumulation and the *CaPAL* expression when the top-ranked RGs were used, including *CaRev05, CaREV08*, and a *CaREV05*/*CaREV08* combination. However, no close correlations were observed between the *CaPAL* expression pattern and the capsaicin content during the fruit development stages when *CaUBI-3* was used for the normalization. Therefore, based on these results, it was considered logical to propose that the top-ranked RGs in this study were appropriate for normalization during pepper fruit development.

It is worth noting that, due to the complexity of the fruit development process, the gene expression analysis of the pepper fruit has now been extended to more precise tissue parts, such as the pericarp, placenta, and even the seeds (Del Rosario Abraham-Juárez et al., 2008; Liu et al., 2013; Phimchan et al., 2014). Therefore, it was believed that the RGs which were identified in this study can be further validated in different tissue sections of the pepper fruit in the future, in particular for experiments which are focused on more specific tissue types.

## Conclusions

In this study, the expression stabilities of previously reported RGs based on RPKM values were evaluated using an entire genome RNA-seq data mining method (Dekkers et al., 2012). Due to their unstable expression patterns, it was determined that most of the RGs were not qualified for normalization. In addition, based on the RNA-seq data sets, 35 novel RGs which were found to be stably expressed during pepper fruit development were selected for further validation using qPCR analyses. These were evaluated using three different statistical algorithms (geNorm, Normfinder, and Boxplot). The results revealed that 10 identified RGs, i.e., *CaREV05, CaREV08, CaREV09, CaREV16, CaREV21, CaREV23, CaREV26, CaREV27, CaREV31* and *CaREV33*, displayed more stable expressions when compared to the traditional RGs used in pepper fruit. Moreover, *CaREV05* and *CaREV08* appeared to be optimal RG combinations if more than one RG was required to improve the accuracy of the qPCR results. In this research study, not only were the optimal RGs in pepper fruit development unveiled, but a new strategy for identifying ideal RG in other sequenced plant species was also established.

## Author contributions

HW and YC: conceived and designed the experiments; YC, XP, JY, QY, RW, ZL, GZ, and ZY: performed the experiments; JY and YC: analyzed the data; and YC, GA and HW: wrote the paper. All of the authors have read and approved the current manuscript.

### Conflict of interest statement

The authors declare that the research was conducted in the absence of any commercial or financial relationships that could be construed as a potential conflict of interest.

## Supplementary material

Supplemental Figure 1 PCR amplification products of the newly identified candidate RGs on the cDNA templates.

Supplemental Figure 2 Specificity of the RG primers demonstrated in the qPCR analysis. Melting curves were generated for all the amplicons.

Supplemental Figure 3 Capsaicin accumulation and expression profiles of the *CaPAL* gene during the pepper fruit development: *CaREV05, CaREV08*, and *CaREV05*/*CaREV08* were used as the RGs for normalization. Also, *CaUBI-3* was used as the RG control.

Supplemental Table 1 **(A)** RPKM values of homologous genes of previously identified RGs against RNA-seq data of different developmental stages of pepper fruit (CM334 and ECW). DPA, Day past anthesis; MG, Mature green; B, breaker. **(B)** RPKM values of homologous genes of previously identified RGs against RNA-seq data of different developmental stages of pepper fruit (ZL-1). length between 1 and 3 cm; F-Dev3:Fruits with length between 3 and 4 cm; F-Dev4:Fruits with length between 4 and 5 cm; F-Dev5:Mature green; The full size green fruit (5–6 cm); F-Dev6:Breaker (fruit turning red); F-Dev7:Breaker plus 3 days; F-Dev8:Breaker plus 5 days; F-Dev9:Breaker plus 7 days.

Supplemental Table 2 **(A)** RPKM values of selected RGs against RNA-seq data of different developmental stages of pepper fruit (CM334 and ECW). DPA, Day past anthesis; MG, Mature green; B, breaker. **(B)** RPKM values of selected RGs against RNA-seq data of different developmental stages of pepper fruit (Zunla-1). F-Dev1, Fruits with length between 0 and 1 cm; F-Dev2, Fruits with length between 1 and 3 cm; F-Dev3, Fruits with length between 3 and 4 cm; F-Dev4, Fruits with length between 4 and 5 cm; F-Dev5, Mature green; The full size green fruit (5–6 cm); F-Dev6, Breaker (fruit turning red); F-Dev7, Breaker plus 3 days; F-Dev8, Breaker plus 5 days; F-Dev9, Breaker plus 7 days.

Supplemental Table 3 Average expression (ME), standard error (STD), and coefficient variation (CV) values of the homologous genes of previously identified RGs and selected RGs. Commone genes between homomogous genes of previously identified RGs and selected RGs were labeled in bold.

Supplemental Table 4 Ct-Values of 35 selected candidate RGs (IM, Immature; MG, Mature Green; B, Breaker; MR, Mature Red; Ave, Average).

Supplemental Table 5 Expression stability values of 35 selected candidate RGs evaluated by geNorm, NormFinder and boxplot.
