# Supplementary figures and images for: Identification of Optimal Reference Genes for Normalization of qPCR Analysis during Pepper Fruit Development
Source: Front Plant Sci. 2017 Jun 29;8:1128. doi: 10.3389/fpls.2017.01128 (PMC5489665; doi:10.3389/fpls.2017.01128)

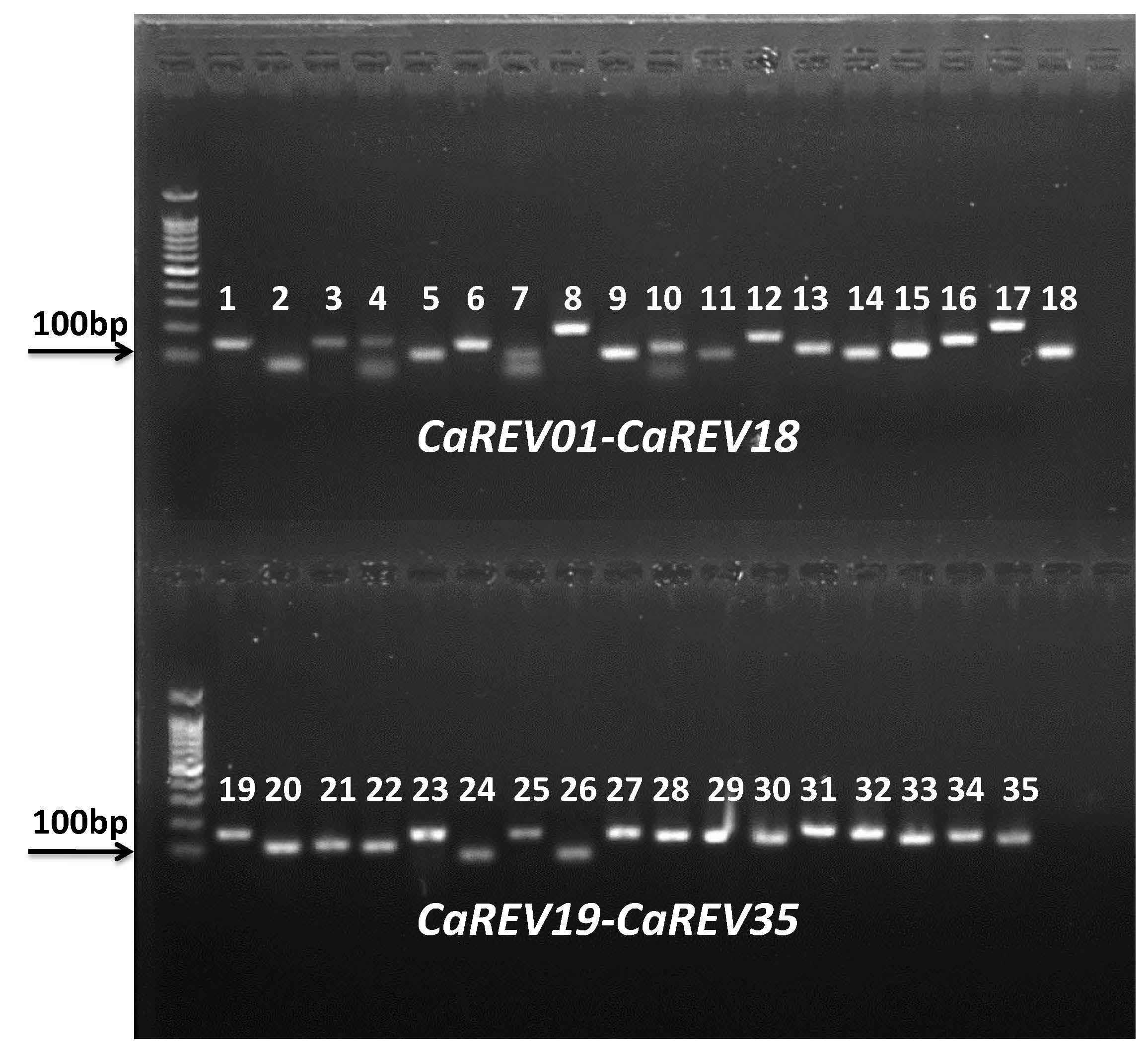

Supplement: Supplemental Figure 2 — Specificity of the RG primers demonstrated in the qPCR analysis. Melting curves were generated for all the amplicons. [file Image2.JPEG]

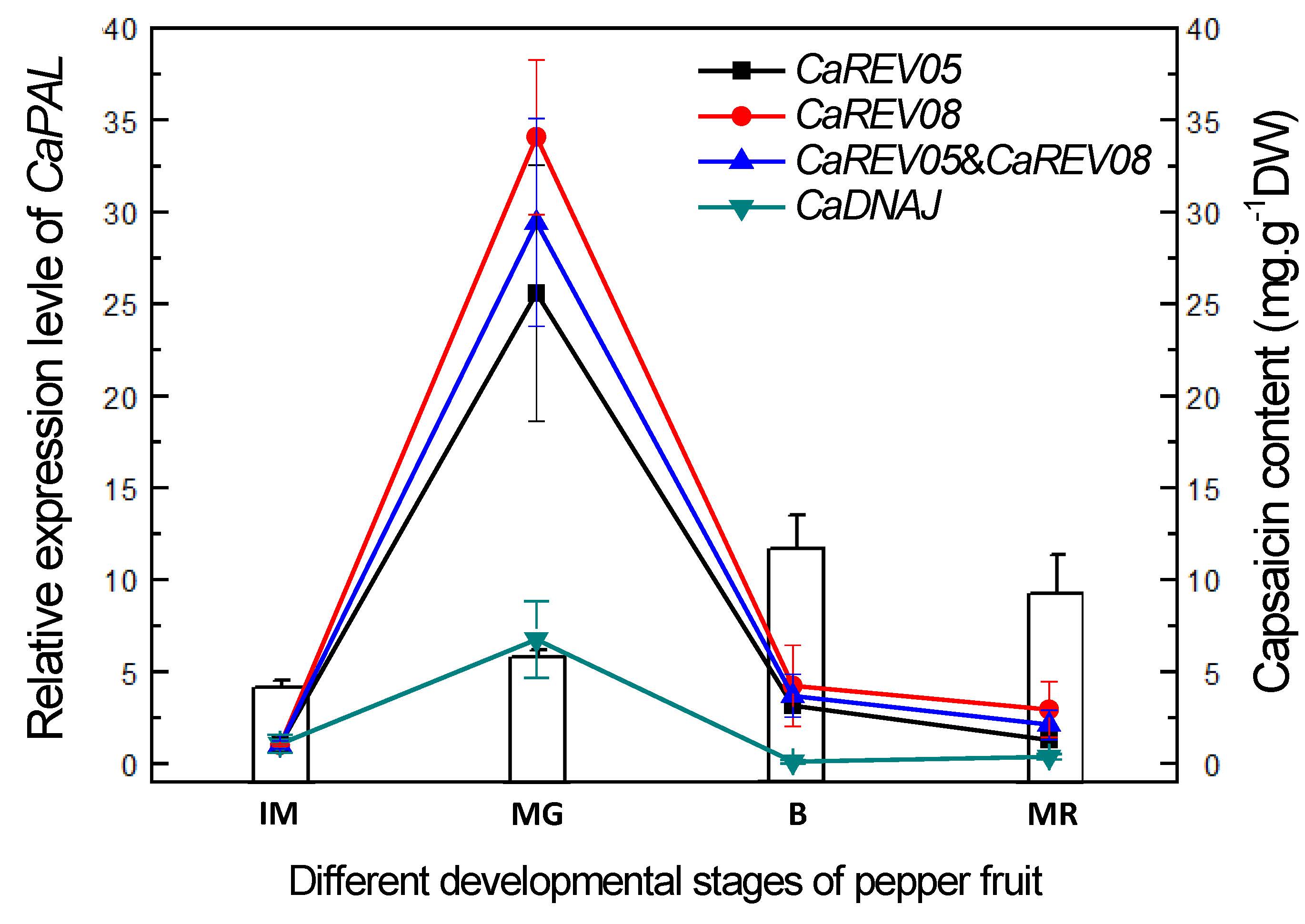

Supplement: Supplemental Figure 3 — Capsaicin accumulation and expression profiles of the CaPAL gene during the pepper fruit development: CaREV05, CaREV08, and CaREV05/CaREV08 were used as the RGs for normalization. Also, CaUBI-3 was used as the RG control. [file Image3.JPEG]
